# Compressive Strength Optimization of 3D-Printed Voronoi Trabecular Bone Using the Taguchi Method

**DOI:** 10.3390/biomimetics11010020

**Published:** 2025-12-31

**Authors:** Suyeon Seo, Ju-Hee Lee, Minchae Kang, Eunsol Park, Min-Woo Han

**Affiliations:** 1 Department of Mechanical Engineering, Dongguk University, 30 Pildong-ro 1, Jung-gu, Seoul 04620, Republic of Korealjh92@dgu.ac.kr (J.-H.L.);; 2 Department of Mechanical, Robotics and Energy Engineering, Dongguk University, 30 Pildong-ro 1, Jung-gu, Seoul 04620, Republic of Korea

**Keywords:** additive manufacturing (AM), 3D printing, Voronoi structure, trabecular bone, fused deposition modeling (FDM), Taguchi method, parameter optimization, compressive strength

## Abstract

The surge in demand for patient-specific orthopedic implants necessitates the precise optimization of design and processing parameters for artificial trabecular bone. This research utilizes Voronoi-based porous structures to replicate the irregular geometry characteristic of natural trabecular bone. All specimens were fabricated through fused deposition modeling (FDM) with polylactic acid (PLA). The study systematically investigated the influence of four primary parameters, namely build orientation, extruder temperature, layer height, and pore count, on compressive strength. To ensure experimental efficiency, the research implemented a Taguchi L20 orthogonal array. Subsequent signal-to-noise (S/N) ratio analysis identified the optimal parameter set as a y-90° build orientation, an extruder temperature of 200 °C, a layer height of 0.2 mm, and a count of 150 pores. These findings underscore the necessity of integrated geometric and process parameter optimization to advance additive manufacturing for orthopedic applications.

## 1. Introduction

Additive manufacturing (AM), also known as 3D printing, has been utilized in the biomedical field, particularly in the development of patient-specific implants [1]. The fabrication of artificial trabecular bones, also known as cancellous bones or spongy bones, offers a promising pathway for personalized orthopedic treatments [2]. While traditional treatments often suffer from limitations in mechanical compatibility and anatomical conformity [3]. AM enables the precise replication of trabecular structures derived from patient-specific computed tomography (CT) data. This capability enhances structural fidelity and biomechanical performance [4]. Such designs not only improve implant stability but also provide a favorable environment for bone ingrowth [5]. However, a significant constraint remains in that patients must provide high-quality CT data in a normal state.

This study focuses on Voronoi-based geometries because their stochastic, cell-like patterns closely resemble natural trabecular morphology. This structural similarity allows Voronoi designs to achieve a balance between porosity and mechanical strength, which confers great potential for the development artificial bones [6,7,8,9,10,11]. To translate these structural advantages into practical applications, the optimization of the 3D printing process is essential. Fused deposition modeling (FDM) is particularly noteworthy due to its cost-effectiveness, material versatility, and capability to fabricate complex geometries [12,13]. Nevertheless, the mechanical performance of FDM-printed components exhibits high sensitivity to process parameters, specifically build orientation, extruder temperature, and layer height. The Taguchi method serves as a robust design approach to optimize these parameters [14,15,16,17]. In contrast to traditional one-variable-at-a-time methods, the Taguchi method utilizes orthogonal arrays to minimize experimental trials while identifying optimal parameter sets [18,19].

Although applications of the Taguchi method to Voronoi-based trabecular scaffolds remain relatively limited, a recent study integrated Taguchi experimental design with gray relational analysis (GRA) to investigate how key processing parameters, namely printing temperature, speed, layer height, and line width, affect compressive strength and elastic modulus. This approach successfully identified optimal conditions that simultaneously improve both mechanical properties [20].

In this research, engineered Voronoi structures are evaluated against natural trabecular bones. While natural bone experiences complex loading scenarios such as fatigue and shear, compressive strength represents the primary determinant for the structural stability of artificial trabecular bones through Voronoi geometries optimized for compressive strength via the Taguchi method. Specifically, the study optimizes four key 3D printing parameters, namely build orientation, extruder temperature, layer height, and the number of pores, to achieve compressive strength comparable to natural trabecular bone. These efforts aim to enhance the structural reliability and biomechanical compatibility of 3D-printed bone scaffolds, thereby establishing a foundation for future clinical applications in personalized orthopedics.

## 2. Materials and Methods

### 2.1. Design and Fabrication

The Voronoi algorithm partitions a plane into discrete regions based on their proximity to specified seed points [6,7,8]. This stochastic cell-like pattern provides a realistic model for the irregular porosity found in natural trabecular bone [9,10,11]. The present study adopts this geometry to achieve structural biomimicry within the scaffold design. To evaluate the mechanical performance of these polylactic acid (PLA) structures systematically, the research utilized the Taguchi method. This approach minimizes the total number of experimental trials while it maintains the capacity to capture interaction effects between multiple variables. An L20 orthogonal array serves as the primary analytical framework for this study.

**Figure 1:**
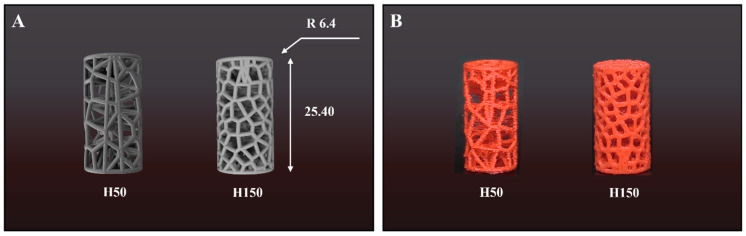
(**A**) CAD models and (**B**) 3D-printed specimens of Voronoi scaffolds with different pore numbers (H50 and H150). Dimensions: Ø 12.8 mm × 25.4 mm.

As illustrated in Figure 1, the specimens follow ASTM D695 standards with a diameter of 12.8 mm and a height of 25.4 mm. The trabecular wall thickness remains constant at 0.4 mm. Quantitative assessments of porosity and strut thickness ensured morphological similarity to human bone during the design phase. Natural trabecular bone typically displays high porosity between 50% and 90%, whereas its thickness varies from 100 to 300 μm.

The Voronoi structures incorporate specific target porosities to remain within the resolution limits of FDM. Specifically, the H50 model employs a porosity of 75.6% and a relative density of 0.2440 to simulate the low-density bone characteristic of the tibia. Conversely, the H150 model possesses a porosity of 54.6% with a relative density of 0.4540 to mimic the denser network of the femur. The strut thickness was fixed at 0.4 mm in the computer-aided design (CAD) model to ensure printability while approximating the upper limit of natural trabecular thickness. This quantitative approach verifies that the artificial scaffolds possess morphological characteristics comparable to actual human bone tissue (Table 1).

PLA was selected as the material for this study because of its versatility and physicochemical properties. Chemically, PLA is an aliphatic polyester synthesized from lactic acid monomers which originate from renewable resources such as corn starch, wheat, and rice. Its chiral nature permits the manipulation of stereochemical configurations (L-lactide and D-lactide) to refine material properties.

In terms of environmental behavior, PLA is widely recognized for its eco-friendly profile. It is biodegradable, recyclable, and compostable, producing non-toxic degradation products. Furthermore, its production requires 25–55% less energy compared to conventional petroleum-based polymers.

Mechanically, the PLA material used in this study demonstrates sufficient stiffness and strength for conceptual bone scaffold modeling. As summarized in Table 2, the material exhibits a density of 1.25 ${g/cm}^{3}$, a Young’s modulus of 3.2 GPa, and a yield strength of 49 MPa. These properties allow the printed Voronoi structures to effectively simulate the mechanical behavior of trabecular bone in reduced-scale experimental setup, providing a reliable basis for optimizing the geometric parameters [21].

The experimental design evaluates four primary factors, namely build orientation, extruder temperature, layer height, and pore count. Build orientation covers five directions: 0°, 45°, and 90° along the *Y*-axis, as well as 45° and 90° along the *Z*-axis. Table 3 details the two levels tested for each remaining variable, while existing literature focus heavily on how temperature and layer height affect the mechanical performance of PLA [22,23,24,25]. Additionally, the tribological properties and coefficient of friction of FDM-printed PLA have been extensively investigated, particularly in relation to filling densities and pattern variations [26,27,28,29].Therefore, this research holds the printing speed and infill density constant to isolate the influence of the chosen parameters. Table 4 shows the L20 Orthogonal Array used in this study.

Although PLA is not a biomaterial, it is chosen as conceptualization for its suitability in structural performance. This study focuses on the structural and mechanical behavior of trabecular bone models; the actual material of the bone is not replicated.

### 2.2. Experiment

**Figure 2:**
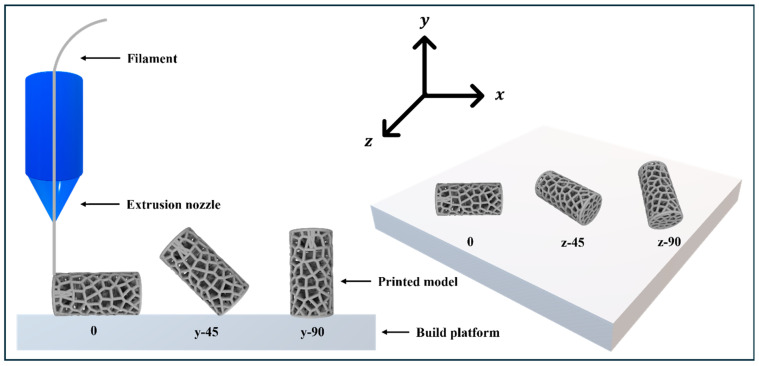
FDM method and build orientations.

Specimens were modeled using Fusion360 and sliced with 3DWOX Desktop software (V1.6.3220) which enabled precise control over printing parameters. A Sindoh 3DWOX 1 printer fabricated the physical samples with PLA filament (Figure 2). To ensure high sample quality and consistency, the slicing parameters remained fixed throughout the fabrication process. These settings included a nozzle diameter of 0.4 mm and a layer height of 0.20 mm. Furthermore, the printing and bed temperatures were maintained at 200 °C and 60 °C, respectively, while a printing speed of 40 mm/s promoted stable deposition for both infills and walls. The internal architecture featured a 15% infill density with a linear raster pattern and a shell thickness of 0.80 mm. To mitigate warping, the fabrication process utilized a raft for enhanced bed adhesion.

Mechanical evaluations involved compression tests conducted on a universal testing machine (OTU-2, Oriental TMCo, OTU-2, Oriental TMCo, Siheung-si, Republic of Korea). The experimental protocol required each specimen to undergo testing three times at room temperature under displacement control, with a crosshead speed maintained at 1.3 mm/min. Finally, the study determined the average compressive strength and the corresponding signal-to-noise (S/N) ratios to assess the structural integrity and performance of the scaffolds.

## 3. Results and Discussion

### 3.1. Signal-to-Noise Ratio Optimization

The Taguchi method categorizes experimental factors into control and noise types. Control factors represent parameters that the designer manipulates directly, whereas noise factors encompass environmental variables, such as temperature and humidity, which remain difficult to regulate [18]. To mitigate the influence of these noise factors and enhance the system robustness, the method utilizes the signal-to-noise (S/N) ratio as a standard performance metric. These ratios follow three distinct classifications based on the target response: “smaller-the-better,” “nominal-the-best,” and “larger-the-better” [30].

Since the present research replicates the compressive strength of natural cancellous bone as accurately as possible, the study adopted the “nominal-the-best” characteristic. This approach allowed for a simultaneous analysis of the S/N ratio and the specific deviations from the target values. The formula used is

(1) $$S/N=10\log\frac{m^{2}}{s^{2}},$$

### 3.2. Theoretical Compressive Strength Based on Gibson–Ashby Model

Theoretical compressive strength values for different pore numbers were calculated using the Gibson–Ashby model [31,32,33,34,35]. The parameters used were $C_{1}$ = 1 [36], $C_{2}$ = 0.3 [37], $n_{1}$ = 2, and $n_{2}$ = 1.5 [36]. Equations (2)–(7) were applied to calculate the mechanical properties, and the results for H50 and H150 are presented in Table 5.

(2) $$\frac{\sigma_{c}}{\sigma_{ys}}=C_{2}{\left(\frac{\rho }{\rho_{S}}\right)}^{n_{2}},$$

(3) Relative Density=1−∅,

(4) $$\rho =\rho_{s}\cdot \left(1-\emptyset \right),$$

(5) $$E=C_{1}\cdot Es\cdot {\left(\frac{\rho }{\rho_{s}}\right)}^{n_{1}},$$

(6) $$\sigma_{y}=C_{2}\cdot \sigma_{ys}\cdot {\left(\frac{\rho }{\rho_{s}}\right)}^{n_{1}},$$

(7) $$\sigma_{c}=\sigma_{y},$$

### 3.3. Experimental Compressive Strength and S/N Analysis

Compression strength was measured through a universal testing machine and each test was repeated three times to ensure the reliability of the average values. Table 6 summarizes the resulting mean compressive strengths and their corresponding S/N ratios for each specimen.

**Figure 3:**
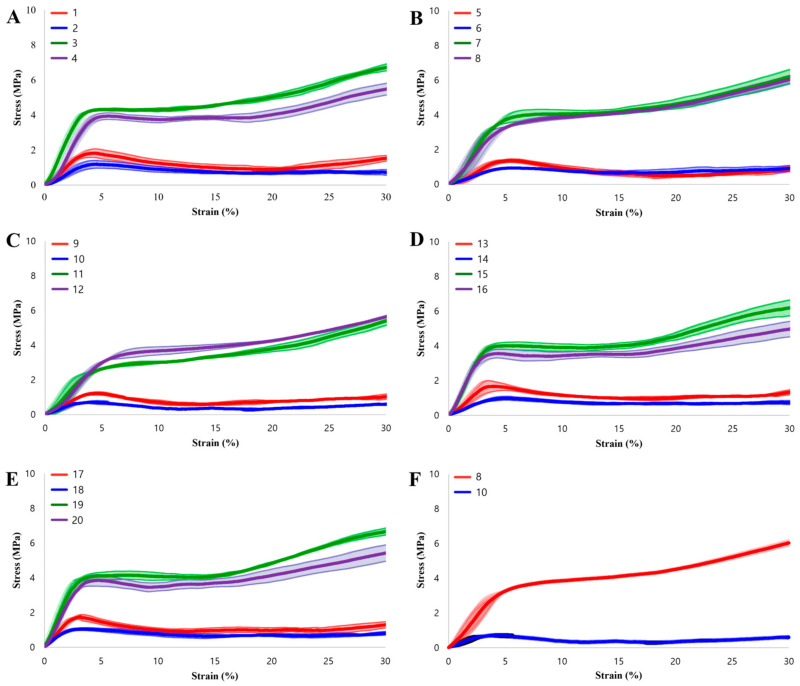
Stress–strain curves of the specimens. (**A**–**E**) Experimental results for all 20 specimens grouped by build orientation. (**F**) Comparison between the optimal performance (Specimen 8) and the worst-case scenario (Specimen 10).

Figure 3 presents the stress–strain curves, which clearly demonstrate the high structural integrity and print precision achieved in this study. Under specific configurations, the experimental data exhibits a strong correlation with theoretical predictions. For example, Specimens 1 and 17 (H50) produced compressive strengths of 1.7866 MPa and 1.7721 MPa, respectively. These figures align closely with the theoretical value of 1.77 MPa. Specimen 8 (H150) recorded a compressive strength of 4.4128 MPa, which validates its theoretical prediction of 4.4970 MPa. This high level of agreement between physical testing and mathematical models confirms the reliability of the Voronoi design approach.

### 3.4. Comparison with Natural Trabecular Bone

Compressive strength determines the biomechanical compatibility and clinical safety of bone-supporting structures [38,39]. While shear and fatigue properties remain critical for long-term implantation, a scaffold must first provide sufficient static load-bearing capacity to support body weight immediately after surgery. Thus, the establishment of optimal parameters for compressive strength represents the essential first step before researchers address multi-axial or cyclic loading conditions. Insufficient strength prevents the structure from supporting body weight and results in deformation or failure. Conversely, excessive strength may induce stress shielding, a phenomenon where natural bone resorption occurs due to load deprivation and ultimately decreases bone density [40,41].

The mechanical response of the optimized PLA structures was evaluated against the target properties of human trabecular bone listed in Table 7. While the raw PLA material possesses a high yield strength of 49 MPa as shown in Table 2, the introduction of the Voronoi porosity effectively modulates these properties to physiological levels. A quantitative analysis of the deviation from target compressive strengths (Table 8) reveals distinct biomimetic capabilities across different bone types.

1. Female Tibia (1.75 ± 1.16 MPa): The H50 structures demonstrated exceptional precision in replicating the female tibia. Specimen 13 achieved a mean strength of 1.7668 MPa, resulting in a negligible deviation of 0.0168 MPa. Similarly, specimen 17 and specimen 1 showed minimal deviations of 0.0221 MPa and 0.0366 MPa, respectively. This confirms that the H50 design parameter is ideal for substituting the female tibia.
2. Female Femur (2.89 ± 1.31 MPa) and Male Tibia (2.59 ± 1.39 MPa): The H150 structures showed suitability for these intermediate load-bearing bones. Specimen 11 (150) recorded a strength of 3.1088 MPa. As detailed in Table 8, this specimen aligns closely with the female femur (deviation of 0.2188 MPa) and the male tibia (deviation of 0.5188 MPa), indicating its versatility for multiple anatomical applications.
3. Male Femur (6.79 ± 2.91 MPa): For the highest load-bearing target, specimen 8 (H150) exhibited the maximum experimental strength of 4.4128 MPa. While it still shows a deviation of 2.3772 MPa from the male femur mean, it falls within the standard deviation range, suggesting potential applicability for specific patient cases requiring moderate load support.

In terms of stiffness, the Gibson–Ashby model predicted a structural Young’s modulus of 191 MPa for H50. Comparing this to the natural bone data in Table 7, the H50 structure aligns well with the elastic modulus range of the female femur (150.89 ± 70.65 MPa) and male femur (360.61 ± 159.40 MPa). This demonstrates that the optimized Voronoi design reduces the high stiffness of the base material (3.2 GPa) to a physiologically relevant range, thereby minimizing the risk of stress shielding while preserving sufficient structural integrity.

The influence of process parameters also proved significant. Specimens fabricated at 220 °C showed higher compressive strength than those produced at 200 °C because higher temperatures enhance inter-layer adhesion. Similarly, specimens with a 0.1 mm layer height outperformed the 0.2 mm variants as finer layering reduces internal defects and improves structural cohesion. Furthermore, specimens with a 0° build orientation displayed the highest strength due to the direct alignment of the load path with the printed layers.

### 3.5. Parameter Effect Analysis and Optimal Conditions

**Figure 4:**
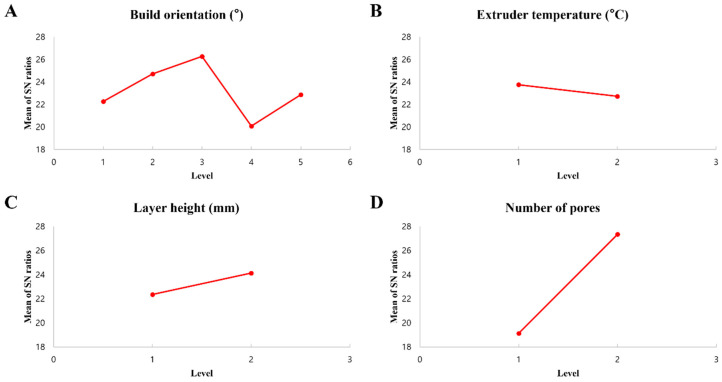
S/N ratios for each process parameter.

The mean S/N ratios for each parameter level are shown in Table 9 and illustrated in Figure 4. Based on the highest S/N values, the optimal parameter combination was identified as A3 (y-90°), B1 (200 °C), C2 (0.2 mm), and D2 (H150). Pore number and build orientation were found to have the most significant impact. This significant dependence is primarily attributed to the inherent anisotropy of FDM fabrication. In FDM parts, mechanical strength is typically superior along the direction of filament deposition (intra-layer) compared to the transverse direction (inter-layer), where structural integrity relies on weaker polymer diffusion bonds. Furthermore, for complex porous geometries like Voronoi scaffolds, the build orientation determines the printing angle of individual struts. Unfavorable orientations can exacerbate the stair-stepping effect on overhanging struts, leading to geometric inaccuracies and stress concentrations that compromise the overall load-bearing capacity [43,44,45,46,47].

To further validate the statistical significance of the experimental results, an Analysis of Variance (ANOVA) was conducted on the S/N ratios. Table 10 presents the ANOVA results, including the degrees of freedom (DF), sum of squares (ss), mean square (MS). F-value, *p*-value, and percent contribution. The *p*-value indicates the statistical significance of each parameter, with a value less than 0.05 typically considered significant at a 95% confidence level.

As shown in Table 10, the number of pores was identified as the most dominant factor, showing a statistically significant effect (p<0.001) and the highest contribution of 56.09%. This confirms that the internal porosity design is the primary determinant of the mechanical performance of the scaffold. The build orientation exhibited the second-highest contribution of 14.86%, aligning with the results, although its *p*-value (0.205) suggests that its effect was less statistically pronounced compared to the pore number within the experimental range. The contributions of extruder temperature and layer height were relatively minor (0.88% and 2.62%, respectively), indicating that these parameters have a limited impact on the S/N ratio variability compared to the geometric design factors.

The optimization of the scaffold design necessitates a critical balance between mechanical strength and biological functionality. While mechanical integrity is essential for load-bearing applications, the pore structure must be sufficiently open to support cellular activities. Previous studies have established that a minimum pore size of 100 μm is necessary for cell migration and nutrient transport, whereas pore sizes ranging from 300 to 600 μm are optimal for vascularization and bone tissue ingrowth.

In this study, the H150 structure was determined based on the optimal design parameter. Although the H150 model features a denser network with a lower porosity (54.6%) compared to the H50 model (75.6%), its stochastic Voronoi pores remain well above the 300 μm biological threshold. This confirms that the H150 design does not compromise biological permeability for the sake of mechanical gain. Instead, it successfully satisfies dual requirements by providing superior mechanical properties comparable to the male femur, while maintaining an interconnected macro-porous architecture conducive to osteoblast infiltration and angiogenesis.

This analysis suggests that not only geometric design but also process optimization is essential for producing mechanically reliable artificial trabecular structures. Build orientation, extruder temperature, layer height, and number of pores all play key roles, and their integrated optimization must be considered in future scaffold design.

## 4. Conclusions

This study successfully evaluated the compressive strength of artificial trabecular bone structures designed with Voronoi geometries. Through the optimization of 3D printing parameters, the resulting scaffolds achieved compressive strengths comparable to those found in natural trabecular bone. The research utilized PLA as the primary filament and employed the Taguchi method to analyze systematically the influence of build orientation, extruder temperature, layer height, and pore count. Experimental results demonstrated that the number of pores and the build orientation represent the most significant factors that govern compressive strength. Specifically, specimens with 150 pores (H150) attained strength levels near those of the human femur, whereas structures with 50 pores (H50) approximated the mechanical properties of the tibia. Furthermore, the experimental data showed strong consistency with theoretical predictions from the Gibson–Ashby model, which validates the efficacy of this structural design approach.

The study identified the optimal parameter combination as a y-90° build orientation, a 200 °C extruder temperature, a 0.2 mm layer height, and a pore count of 150. This specific configuration maximizes structural cohesion, inter-layer adhesion, and overall mechanical integrity. These findings underscore the necessity to optimize both geometric design and process parameters simultaneously to develop reliable bone scaffolds. While PLA served as a suitable material for printability and initial mechanical validation, future research should explore bioresorbable and biocompatible materials, such as PCL, PLLA, and β-TCP reinforced composites, to enhance clinical applicability. Subsequent studies should also evaluate dynamic behaviors, including fatigue properties and shear resistance, alongside biological responses and in vivo performance to advance toward personalized orthopedic solutions. In conclusion, this research establishes a robust framework for the efficient fabrication of mechanically optimized, patient-specific artificial trabecular bone. These findings provide a solid foundation for future advancements in regenerative medicine and orthopedic implant design.

## Figures and Tables

**Table 1:** Design parameters of Voronoi structures.

Number of Pores	Distance (mm)	Thickness (mm)	Porosity (%)
50	0.1	0.4	75.6
150	0.1	0.4	54.6

**Table 2:** PLA properties.

Property	PLA
$\mathrm{Density}\;{(\rho }_{s})$	$1.25\;{g/cm}^{3}$
Young’s modulus (Es)	3.2 GPa
$\mathrm{Yield}\;\mathrm{strength}\;{(\sigma }_{ys})$	49 MPa

**Table 3:** Parameters and their levels.

Parameter	Level				
	1	2	3	4	5
A: Build orientation (°)	0° (flat)	45° on *Y*-axis	90° on *Y*-axis (On-edge)	45° on *Z*-axis	90° on *Z*-axis
B: Extruder temperature (°C)	200	220	-	-	-
C: Layer height (mm)	0.1	0.2	-	-	-
D: Number of pores	H50	H150	-	-	-

**Table 4:** L20 Orthogonal Array.

Num	A	B	C	D
1	0	200	0.1	H50
2	0	220	0.2	H50
3	0	200	0.2	H150
4	0	220	0.1	H150
5	y-45	200	0.1	H50
6	y-45	220	0.2	H50
7	y-45	200	0.2	H150
8	y-45	220	0.1	H150
9	y-90	200	0.1	H50
10	y-90	220	0.2	H50
11	y-90	200	0.2	H150
12	y-90	220	0.1	H150
13	z-45	200	0.1	H50
14	z-45	220	0.2	H50
15	z-45	200	0.2	H150
16	z-45	220	0.1	H150
17	z-90	200	0.1	H50
18	z-90	220	0.2	H50
19	z-90	200	0.2	H150
20	z-90	220	0.1	H150

**Table 5:** Theoretical properties of specimens by number of pores.

Property	H50	H150
Relative density	0.2440	0.4540
Porous structure density (ρ)	$0.3050\;{g/cm}^{3}$	$0.5675\;{g/cm}^{3}$
Young’s modulus (E)	0.1910 GPa	0.6590 GPa
$\mathrm{Yield}\;\mathrm{strength}\;{(\sigma }_{y})$	1.7700 MPa	4.4970 MPa
$\mathrm{Compressive}\;\mathrm{strength}\;{(\sigma }_{c})$	1.7700 MPa	4.4970 MPa

**Table 6:** Compressive strength and S/N ratio.

Num	Mean Compressive Strength (MPa)	The Standard Deviation of the Mean Value	S/N Ratio (dB)
1	1.7866	0.2354	17.6050
2	1.1859	0.2356	14.0392
3	4.2774	0.1198	31.0516
4	4.0544	0.1940	26.4047
5	1.3827	0.1341	20.2659
6	0.9424	0.0665	23.0235
7	4.1911	0.2205	25.5853
8	4.4128	0.1397	29.9882
9	1.2208	0.1169	20.3748
10	0.7099	0.0752	19.5042
11	3.1088	0.0489	36.0737
12	3.7623	0.1311	29.1293
13	1.7668	0.3757	13.4471
14	0.9783	0.1124	18.7907
15	3.9631	0.2205	25.0913
16	3.6362	0.2570	23.0152
17	1.7721	0.1391	22.1053
18	1.0778	0.0838	22.1856
19	4.0425	0.2031	25.9800
20	3.9849	0.3473	21.2116

**Table 7:** Compressive strength of human trabecular bone [42].

Sex	Bone	Compressive Strength (MPa)	Elastic Modulus (MPa)
Male	Femur	6.79 ± 2.91	360.61 ± 159.40
	Tibia	2.59 ± 1.39	108.80 ± 52.88
Female	Femur	2.89 ± 1.31	150.89 ± 70.65
	Tibia	1.75 ± 1.16	73.45 ± 55.06

**Table 8:** Deviation from target compressive strength.

Num	Deviation from the Target Value			
	Male Femur (6.79 ± 2.91 MPa)	Male Tibia (2.59 ± 1.39 MPa)	Female Femur (2.89 ± 1.31 MPa)	Female Tibia (1.75 ± 1.16 MPa)
1	5.0034	0.8034	1.1034	0.0366
2	5.6041	1.4041	1.7041	0.5641
3	2.5126	1.6874	1.3874	2.5274
4	2.7351	1.4650	1.1650	2.3050
5	5.4073	1.2073	1.5073	0.3673
6	5.8476	1.6476	1.9476	0.8075
7	2.5963	1.6037	1.3037	2.4437
8	2.3772	1.8228	1.5228	2.6628
9	5.5692	1.3692	1.6692	0.5292
10	6.0801	1.8801	2.1801	1.0401
11	3.6813	0.5188	0.2188	1.3588
12	3.0277	1.1723	0.8723	2.0123
13	5.0232	0.8232	1.1232	0.0168
14	5.8117	1.6117	1.9117	0.7717
15	2.8270	1.3731	1.0731	2.2131
16	3.1539	1.0462	0.7462	1.8862
17	5.0179	0.8179	1.1179	0.0221
18	5.7122	1.5122	1.8122	0.6722
19	2.7475	1.4525	1.1525	2.2925
20	2.7974	1.4026	1.1026	2.2426

**Table 9:** S/N ratios by parameter level.

Level	Build Orientation	Extruder Temperature	Layer Height	Number of Pores
1	22.2751	23.7580	22.3547	19.1341
2	24.7157	22.7292	24.1325	27.3531
3	26.2705	-	-	-
4	20.0861	-	-	-
5	22.8706	-	-	-
Delta	6.1844	1.0288	1.7778	8.2190
Ranking	2	4	3	1

**Table 10:** Analysis of Variance (ANOVA) for S/N ratios.

	DF	Adj SS	Adj MS	F-Value	*p*-Value	Contribution (%)
Build orientation	4	89.51	22.38	1.75	0.204550	14.86
Extruder temperature	1	5.29	5.29	0.41	0.532547	0.88
Layer height	1	15.80	15.80	1.23	0.288533	2.62
Number of pores	1	337.76	337.76	26.36	0.000247	56.09
Residual error	12	153.77	12.81	-	-	25.54
Total	19	602.13	-	-	-	100

## Data Availability

The original contributions presented in the study are included in the article; further inquiries can be directed to the corresponding authors.

## Abbreviations

The following abbreviations are used in this manuscript:

AM	Additive manufacturing
FDM	Fused deposition modeling
PLA	Polylactic acid
S/N	Signal-to-noise
CT	Computed tomography
GRA	Gray relational analysis
CAD	Computer-aided design
